# Circulating Autoantibodies in Age-Related Macular Degeneration Recognize Human Macular Tissue Antigens Implicated in Autophagy, Immunomodulation, and Protection from Oxidative Stress and Apoptosis

**DOI:** 10.1371/journal.pone.0145323

**Published:** 2015-12-30

**Authors:** Alessandro Iannaccone, Francesco Giorgianni, David D. New, T. J. Hollingsworth, Allison Umfress, Albert H. Alhatem, Indira Neeli, Nataliya I. Lenchik, Barbara J. Jennings, Jorge I. Calzada, Suzanne Satterfield, Dennis Mathews, Rocio I. Diaz, Tamara Harris, Karen C. Johnson, Steve Charles, Stephen B. Kritchevsky, Ivan C. Gerling, Sarka Beranova-Giorgianni, Marko Z. Radic, Health ABC study

**Affiliations:** 1 Department of Ophthalmology, Hamilton Eye Institute, University of Tennessee Health Science Center, Memphis, TN, United States of America; 2 Department of Pharmaceutical Sciences, University of Tennessee Health Science Center, Memphis, TN, United States of America; 3 Department of Microbiology, Immunology & Biochemistry, University of Tennessee Health Science Center, Memphis, TN, United States of America; 4 Department of Internal Medicine/Endocrinology, University of Tennessee Health Science Center, Memphis, TN, United States of America; 5 Charles Retina Institute, Memphis, TN, United States of America; 6 Department of Preventive Medicine, University of Tennessee Health Science Center, Memphis, TN, United States of America; 7 Eye Specialty Group, Memphis, TN, United States of America; 8 Southern College of Optometry, Memphis, TN, United States of America; 9 National Institute on Aging, NIH, Bethesda, MD, United States of America; 10 Sticht Center on Aging, Wake Forest University, Winston-Salem, NC, United States of America; University of Florida, UNITED STATES

## Abstract

**Background:**

We investigated sera from elderly subjects with and without age-related macular degeneration (AMD) for presence of autoantibodies (AAbs) against human macular antigens and characterized their identity.

**Methods:**

Sera were collected from participants in the Age-Related Maculopathy Ancillary (ARMA) Study, a cross-sectional investigation ancillary to the Health ABC Study, enriched with participants from the general population. The resulting sample (mean age: 79.2±3.9 years old) included subjects with early to advanced AMD (n = 131) and controls (n = 231). Sera were tested by Western blots for immunoreactive bands against human donor macular tissue homogenates. Immunoreactive bands were identified and graded, and odds ratios (OR) calculated. Based on these findings, sera were immunoprecipitated, and subjected to 2D gel electrophoresis (GE). Liquid chromatography-tandem mass spectrometry (LC-MS/MS) was used to identify the targets recognized by circulating AAbs seen on 2D-GE, followed by ELISAs with recombinant proteins to confirm LC-MS/MS results, and quantify autoreactivities.

**Results:**

In AMD, 11 immunoreactive bands were significantly more frequent and 13 were significantly stronger than in controls. Nine of the more frequent bands also showed stronger reactivity. OR estimates ranged between 4.06 and 1.93, and all clearly excluded the null value. Following immunoprecipitation, 2D-GE and LC-MS/MS, five of the possible autoreactivity targets were conclusively identified: two members of the heat shock protein 70 (HSP70) family, HSPA8 and HSPA9; another member of the HSP family, HSPB4, also known as alpha-crystallin A chain (CRYAA); Annexin A5 (ANXA5); and Protein S100-A9, also known as calgranulin B that, when complexed with S100A8, forms calprotectin. ELISA testing with recombinant proteins confirmed, on average, significantly higher reactivities against all targets in AMD samples compared to controls.

**Conclusions:**

Consistent with other evidence supporting the role of inflammation and the immune system in AMD pathogenesis, AAbs were identified in AMD sera, including early-stage disease. Identified targets may be mechanistically linked to AMD pathogenesis because the identified proteins are implicated in autophagy, immunomodulation, and protection from oxidative stress and apoptosis. In particular, a role in autophagy activation is shared by all five autoantigens, raising the possibility that the detected AAbs may play a role in AMD via autophagy compromise and downstream activation of the inflammasome. Thus, we propose that the detected AAbs provide further insight into AMD pathogenesis and have the potential to contribute to disease biogenesis and progression.

## Introduction

Age-related macular degeneration (AMD) is a highly prevalent, multifactorial, polygenic and complex retinal degenerative disease [1, 2] in which characteristic deposits, termed drusen, develop mostly under the retinal pigment epithelium (RPE) at the interface with subjacent choroidal circulation, and clinically visible RPE changes (i.e., migration and clustering, appearing clinically as macular focal hyperpigmentation, and loss, resulting in focal drop-out, appearing clinically as hypopigmentation) occur [3–6]. It is estimated that approximately one in three elderly over the age of 75 develops early AMD, which progresses to advanced AMD and affects approximately 10% of the elderly in this age range. With early AMD, vision loss is still minimal [1]. In advanced AMD, choroidal neovascularization (nvAMD) and/or patches of RPE loss [geographic atrophy (GA)] develop, leading to photoreceptor and severe central vision loss, which often results in legal blindness and significant compromise of the quality of life of affected patients. Characterization of drusen and RPE changes helps predict likelihood of disease progression [7–11], but the underlying mechanisms leading up to these changes and to AMD progression remain incompletely understood. Thus, identification of factors and mechanisms that favor the development of AMD and its progression from early to advanced AMD would prove invaluable in mitigating the impact of AMD on the elderly.

It is now widely accepted that inflammation and the immune system play important roles in AMD pathogenesis [12–26]. In AMD, the choriocapillaris and the RPE are the object of antibody (Ab)-mediated complement deposition, and accumulating immune complexes have been shown to contribute to RPE degeneration, and drusen formation [3, 27]. Dendritic cells (DCs) infiltrate the Bruch’s membrane (BM), project processes inside drusen cores, and break the blood-retinal barrier, which leads to RPE loss due to inflammatory damage. RPE loss coincides with, and is proportional to drusen formation [3]. Drusen may act as a reservoir of autoantigens that, upon presentation by DC and other immunocompetent cells, may drive autoimmune-mediated damage to macular tissues [3]. Thus, inflammatory and immune-mediated events are likely at play in drusen biogenesis and RPE loss in AMD.

Accordingly, genetic factors linked to inflammation have been clearly associated both with probability of having AMD and progressing to advanced AMD [28–48]. Thus, genetic evidence argues that AMD can be considered at least in part as a congenital predisposition to defective modulation of inflammation with late-onset manifestations, likely modulated by a number of intervening factors. While statistical approaches have consistently confirmed the role of genetic factors in AMD [39, 49–51], when population samples without clear-cut, preexisting advanced AMD are included in the analyses, these methods do not achieve ideal discrimination of AMD even when modifiable environmental factors are accounted for [44]. Therefore, AMD routine genotyping is not presently recommended [52]. Gene-environment interactions have also been demonstrated [37, 53–55], highlighting the complexity of AMD. Consistent with this complexity, studies have shown that genomic biomarkers in combination with autoimmune serum biomarkers such as anti-carboxyethylpyrrole (CEP) auto-Abs (AAbs) are better predictors of AMD than genomic biomarkers alone [56]. Thus, there is much value in characterizing further the autoimmune component of AMD.

We have proposed that AAbs could be very useful biomarkers and play a mechanistic role in AMD [57]–a view that is shared by others [12–14, 17, 24, 56, 58, 59]. Here, we present evidence for AAbs against human macular tissue antigens in AMD sera also in participants with early disease stages, and we identify five macular autoantigens, all of which are potentially related to AMD pathogenesis. These findings raise the possibility that these AAbs are not mere after-the-fact biomarkers but, in fact, active contributors to disease development and progression.

## Methods

### Participant Population

All investigations were conducted in compliance with the Declaration of Helsinki and following approval by the Institutional Review Board of the University of Tennessee Health Science Center. Our study involved primarily participants in the Health ABC Age-Related Maculopathy Ancillary (ARMA) Study, and details about its participants have been published [60–63]. In brief, the resulting study population included 362 participants (mean age: 79.2 ± 3.9 years old; range: 63–91 years old; 54% females; 81% self-reported Whites), 131 of whom with AMD and 231 unaffected. Based on the original AREDS classification [7], 92 AMD cases were early to intermediate stage (category 3), and 39 advanced (category 4), 70% of which nvAMD. All participants were chosen by design to be free of diabetes >2 year duration and of diabetic retinopathy, glaucoma, inflammatory eye diseases, retinal conditions other than AMD that could confound the study (e.g., retinal detachment), systemic autoimmune conditions, or any other condition that was deemed a potential confounder. Further details are provided in S1 Supporting Information.

After obtaining written informed consent, serum samples were collected from all eligible participants, stored at –80°C according to standard methods, and subsequently analyzed by Western blots (WBs). A subset of serum aliquots also underwent immunoprecipitation (IP) and 2D gel electrophoresis (2D-GE). Putative IP autoantigens were identified via liquid chromatography tandem mass spectrometry (LC-MS/MS) and confirmed by ELISA.

### Human Tissue Harvesting, Western Blotting and Immunoreactivity Analysis

Sera were assessed for reactivity against homogenates comprising neuroretina (nRet), RPE, BM and choroid (Ch), harvested from the macular region of normal human donor eyes (≥60 yo) [64], as detailed in S1 Supporting Information and in **S1 Fig**. Human donor eyes used in these experiments were obtained from cadaver eyes by the Mid-South Eye Bank, Memphis, TN, and by the National Disease Research Interchange (NDRI), Philadelphia, PA, and provided to us as anonymous specimens. Homogenates from various donor eyes were pooled, and used as source of antigens for WBs. Details on the WB methodology are also provided in S1 Supporting Information. In brief, three films were generated for each blot with exposure times of 5, 15, and 30 sec. Films were then evaluated to determine the presence of bound Ab in a semi-quantitative manner and graded. WBs were graded by trained lab staff for presence and intensity of immunoreactive (IR) bands. To minimize bias, staff was masked to disease status of the samples. Details on the methods and technique used to grade the WB gels are provided in S1 Supporting Information. The method was developed and tested for reproducibility, based on a conceptually similar scale previously developed to assess other images [65] (see **S1 Table and S2 Fig**). IR values were recorded in a database and analyzed as described in S1 Supporting Information.

### Immunoprecipitation (IP), 2D Gel Electrophoresis (2D-GE) and Liquid Chromatography Tandem Mass Spectrometry (LC-MS/MS)

A representative subset of AMD (n = 18) and control samples (n = 15) was used to identify candidate protein autoantigens that are preferentially targeted by AAbs from AMD sera. All AMD subjects in this subset had at least one eye with AREDS stage 3 disease [7], and half of them had at least one eye with advanced AMD. Control subjects included AREDS stages 0–2 [7]. Macular tissue homogenates were immunoprecipitated with AMD and control sera. The human macular antigens pulled down by IP from these AMD and control sera were separated on 2D-GE gels, stained by SYPRO-Ruby Gel Stain (BioRad) and analyzed with Progenesis SameSpots software (Nonlinear Dynamics, USA). This software not only aligns images, matches the same spots, and finds differentially expressed protein spots, but also adjusts for the loading differences across the set of gels by normalizing spot intensities to total gel stain intensity. Normalized spot volumes (quantity) are also calculated automatically by the software. 2DGE is a semi-quantitative method that determines the absolute levels of auto-reactivity. This semi-quantitative information from this discovery experiment was used to guide decisions on which proteins should be evaluated by quantitative assays (ELISA, see below) performed on the proteins identified by LC-MS/MS.

The proteins of interest were digested with trypsin as described previously [66] [67], and the digests were analyzed with LC-MS/MS performed on an ion trap tandem mass spectrometer. The proteins were identified via searches of the UniProt protein sequence database. Further details are provided below and in S1 Supporting Information.

### Enzyme-Linked Immunosorbent Assays (ELISA) for confirmation of specific autoreactivities against detected antigens by circulating auto-antibodies

Aliquots of the serum samples used in the 2D-GE and LC-MS/MS experiments were used in ELISA to test their reactivity against recombinant proteins corresponding to the candidate target proteins identified by LC-MS/MS (see results). Immobilon IV 96-well plates (Millipore) were coated with recombinant human proteins and probed in duplicate with serum from either normal or AMD subjects for 2 hrs at room temperature. Plates were then washed and probed with HRP-conjugated anti-human IgG. Again, control subjects included AREDS stages 0–2 and all sera were from AMD participants grade ≥3 based on the original AREDS grading [7]. This selected sub-group of AMD participants had an AMD score of ≥4 based on the new simplified AREDS scoring system [8], and of 7–9 or higher (central GA and/or neovascular AMD) based on the new AREDS severity scale [9]. ELISA reactivities were measured with a microQuant Spectrophotometer (BioTek) and expressed as optical density values (OD450).

### Statistical analyses

Each serum sample generated 100 data points (thus, 13,100 data points for the AMD serum sample group, and 23,100 for the control group). We accounted for multiple comparisons using the Benjamini-Hochberg (B-H) multiple test correction and p<0.05 [68, 69], as shown by Thissen et al [16]. In brief, the B-H correction limits the type-I error, or false discovery rate (FDR). Starting from an initial α = 0.05 cut-off, the B-H method allows for application of sequentially more stringent α values for each of the comparisons made until the observed p-value exceeds the critical B-H value (which, for our dataset was 0.0065). This method, utilized with large data arrays [70], avoids the overcorrection (and, thus, the risk of Type-II error) caused when classical methods (e.g., Bonferroni) are applied to exploratory datasets that require multiple comparisons.

Two levels of preplanned analysis were performed by comparing the entire AMD and control dataset via unpaired t-test for samples with unequal variances and by constructing 2x2 tables and computing odds ratios (ORs), 95% confidence intervals (CI), χ^2^ statistic, and p-values, for each significant IR band. Differences were ranked from the highest χ^2^ statistic to the lowest and the B-H multiple test correction method was applied until the significance cut-off defined by this method was no longer significant. Only more frequent and/or more intense IR bands meeting the B-H criteria were considered significant.

Sera exhibiting IR bands meeting these criteria were considered more likely to contain AAbs and were further analyzed by IP, 2D-GE and LC-MS/MS to identify potential autoantigens. Differences in 2D-GE spot intensity between control and AMD sera were quantified automatically by the Progenesis SameSpots software via one-way analysis of variance (ANOVA). To identify differentially abundant proteins, LC-MS/MS datasets were used to interrogate the UniProt protein sequence database (subset of human proteins) using the SEQUEST HT search engine (Proteome Discoverer 1.4 software suite, Thermo Scientific). Significance of the peptide spectrum matches was assigned via Percolator, a standard algorithm for LC-MS/MS data, using a threshold value of q = 0.01, which corresponds to an FDR of 1% for peptide match assignment. Additional details about this procedure are provided in S1 Supporting Information.

Lastly, ELISA reactivities were compared by means of 2-tailed, unpaired Student’s t-test (or equal or unequal variances, as appropriate) with α = 0.05 as cut-off for statistical significance also for these comparisons.

## Results

**Fig 1:**
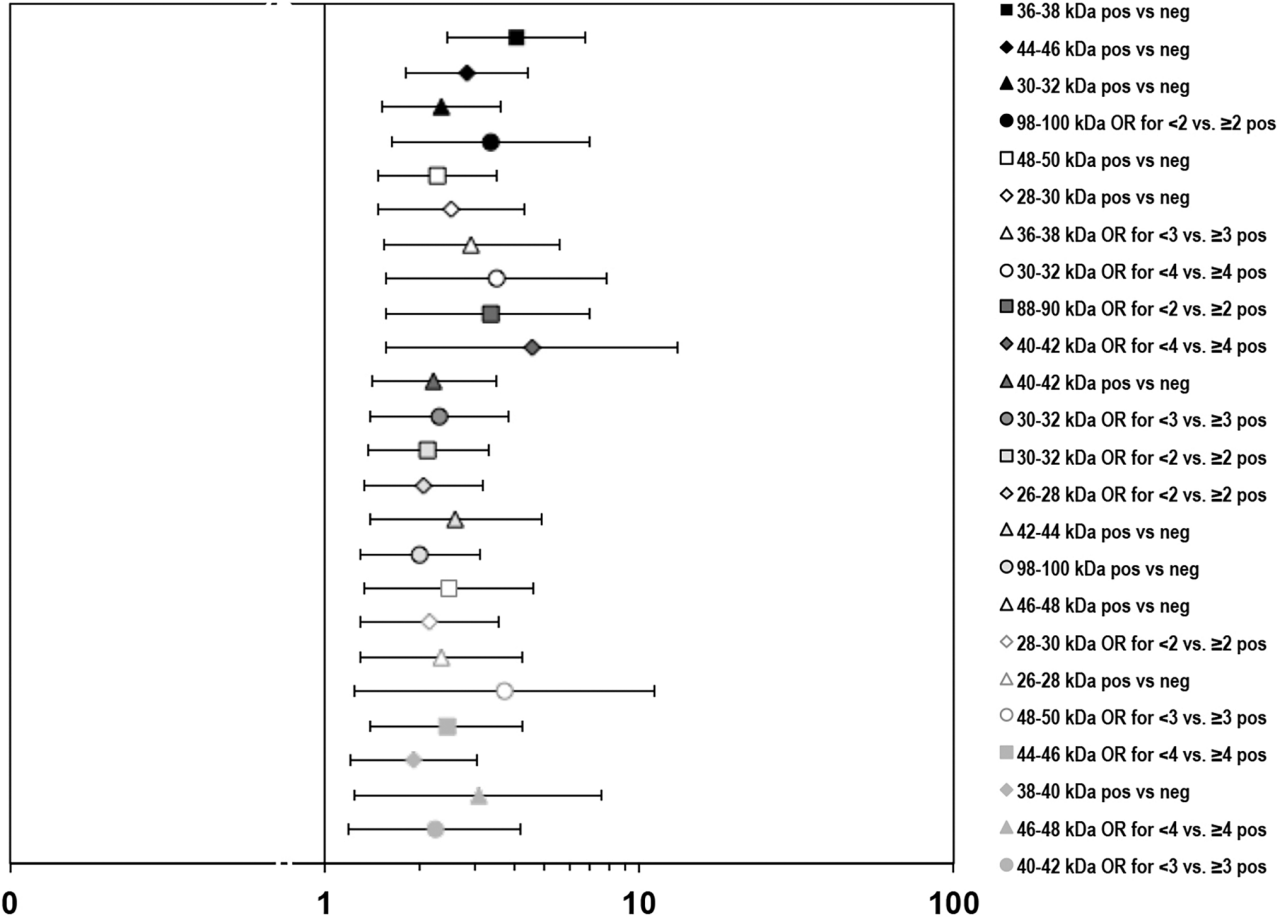
Plot of odds ratios (ORs) and 95% CIs for immunoreactive (IR) bands detected by Western blot. OR data points are ordered, top to bottom, from the highest χ^2^ statistic (32.46, p = 0.012 x 10^−6^) to the lowest (7.30, p = 0.0068) meeting the critical B-H value. All IR bands listed were significantly more intense and/or more common in AMD sera.

AMD sera recognized a greater number of IR intervals of human macular antigens and exhibited stronger autoreactivity compared to control sera. More frequent IR was observed in AMD for the dataset as a whole (p = 0.02 x 10^−8^) and, after applying the B-H multiple test correction method to our data set, specifically for 11 distinct 2-kDa IR intervals. The more frequent IR bands clustered primarily in two areas, between 26 and 32 kDa (n = 3), between 36 and 50 kDa (n = 7), and at the 98–100 kDa interval. By the same method, greater IR intensity was observed for 13 levels of comparisons among the observed IR bands. Of these, 9 of 11 bands coincided with the intervals in which IR was also seen more frequently. Some bands were significantly more intense for various cut-off levels of comparison (e.g., the 40–42 kDa band showed greater IR for the <3 vs. ≥3 and <4 vs. ≥4 IR score comparisons). Two comparisons were significant uniquely for frequency of the IR band (38–40 kDa) and for IR intensity (88–90 kDa, <2 vs. ≥2 comparison), respectively. **Fig 1** illustrates these results, by presenting point estimates of ORs and 95% CIs for the IR bands whose χ^2^ values met the B-H critical value, ranked by the latter. The lowest significance level that met the B-H critical value was a χ^2^ of 7.85, p = 0.0051. All IR intervals meeting the B-H criteria for inclusion were highly significant, with the 95% CIs excluding clearly the null (OR = 1.0) value and OR values ranging between 1.93 and 4.56.

**Fig 2:**
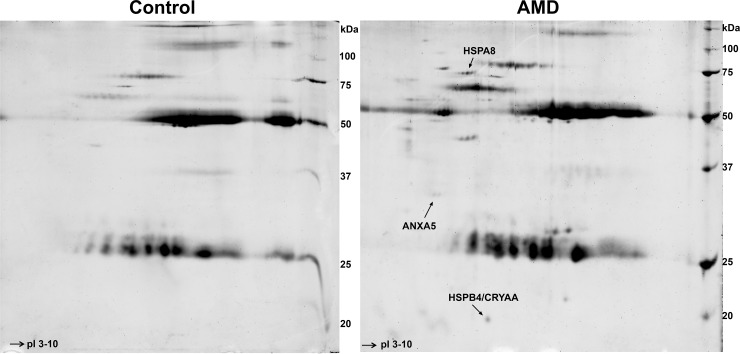
Example of two dimensional gel electrophoresis (2D-GE) analyses comparing control vs. AMD sera. The left panel shows a gel on which protein from human macular lysate immunoprecipitated by a control serum sample have been separated on two dimensions (by molecular weight and pI), compared to an AMD sample ran identically and simultaneously shown on the right. There are several spots on the 2D-GE AMD gel that appear different compared to the control one. Confirmed antigens recognized by the AAbs present in the serum of this one particular AMD participant included HSPA8, ANXA5 and HSPB4/CRYAA (arrows). See text for further discussion of these findings. Note also how the AMD gel exhibits a stronger “load” compared to the control gel, consistent with the fact that AMD sera tended to be more often autoreactive and thus, even when some autoreactivities were shared, AMD sera exhibited much stronger levels of reactivity, indicative of a larger amount and numbers of proteins pulled down by the IP process.

AMD sera with the greatest and most frequent IR and controls were used to conduct additional IP, 2D-GE and LC-MS/MS experiments, which identified five potential targets of autoreactivity **(Table 1)**: two members of the heat shock protein 70 (HSP70) family, HSPA8 and HSPA9; another member of the HSP family, HSPB4, also known as alpha-crystallin A chain (CRYAA); Annexin A5 (ANXA5); and Protein S100-A9 (S100A9), also known as calgranulin B that, when complexed with S100A8, forms calprotectin. LC-MS/MS spectra for some of the identified peptides are also illustrated in **S3 Fig.** A representative pair of gels illustrating an example of the differentially expressed spots on 2D-GE is shown in **Fig 2.** In this example, three of the aforementioned targets– HSPA8, ANXA5, and HSPB4 (CRYAA)–were confirmed among the immunoprecipitated macular lysate proteins that were upregulated in the AMD serum compared to the control serum and subsequently verified as such by ELISA criteria (see below). Note that additional spots that appear to be upregulated on 2D-GE for this one AMD serum are present. While it is possible that these spots too may represent meaningful autoreactivities, they were not confirmed as significant in the remaining subset of AMD samples subjected to 2D-GE and/or by ELISA testing and, thus, have not been considered further. Note also the different intensity of the autoreactivity for the three targets: HSPA8 is highest in this case of AMD, whereas ANXA5 and HSPB4/CRYAA were weaker. Other AMD sera (see below) exhibited much stronger reactivity for ANXA5 or for other targets. This inter-subject variability emphasizes the concept, proposed by Adamus et al. [59], of different “AAb signatures” that AMD patients may have.

**Table 1:** Summary of autoantigens identified by LC-MS/MS.

Gene Name	Protein denomination	UniProt Accession No.	Peptides Identified ^a^	XCorr (z) ^b^
**HSPA8**	Heat shock cognate 71 kDa protein, a.k.a. HSP70-8	P11142	NQTAEKEEFEHQQK	3.34 (3)
			RFDDAVVQSDM*K	3.41 (2)
			M*KEIAEAYLGK	3.94 (2)
			RFDDAVVQSDMK	3.37 (2)
			NQVAM*NPTNTVFDAK	5.19 (2)
			MKEIAEAYLGK	3.35 (2)
			SQIHDIVLVGGSTR	4.84 (2)
			HWPFM*VVNDAGRPK	3.44 (3)
			NSLESYAFNM*K	4.27 (2)
			MVNHFIAEFK	2.92 (2)
			NQVAMNPTNTVFDAK	4.83 (2)
			NSLESYAFNMK	3.40 (2)
			SFYPEEVSSM*VLTK	5.06 (2)
			TVTNAVVTVPAYFNDSQR	4.83 (3)
			GPAVGIDLGTTYSCVGVFQHGK	4.65 (3)
			SFYPEEVSSMVLTK	4.05 (2)
			SINPDEAVAYGAAVQAAILSGDK	7.11 (2)
**HSPA9**	Stress-70 protein, mitochondrial, a.ka. GRP75	P38646	VQQTVQDLFGR	3.48 (2)
			DAGQISGLNVLR	3.34 (2)
**HSPB4**	AlphaA-crystallin or CRYAA	P02489	VQDDFVEIHGK	3.72 (3)
			TVLDSGISEVR	4.39 (2)
**ANXA5**	Annexin A5	P08758	LYDAYELK	2.99 (2)
			VLTEIIASR	3.45 (2)
			GTVTDFPGFDER	3.82 (2)
			M*LVVLLQANR	3.79 (2)
			NFATSLYSM*IK	3.20 (2)
			YM*TISGFQIEETIDR	5.23 (2)
			SIPAYLAETLYYAM*K	4.20 (2)
			GLGTDEESILTLLTSR	5.61 (2)
			ETSGNLEQLLLAVVK	3.69 (2)
**S100A9**	S100 calcium-binding protein A9, a.k.a. calgranulin B	P06702	VIEHIMEDLDTNADK	4.91 (2)
			NIETIINTFHQYSVK	4.98 (2)

^a^ Asterisk denotes oxidized methionine

^b^ Cross correlation score (XCorr) and charge (z)

**Fig 3:**
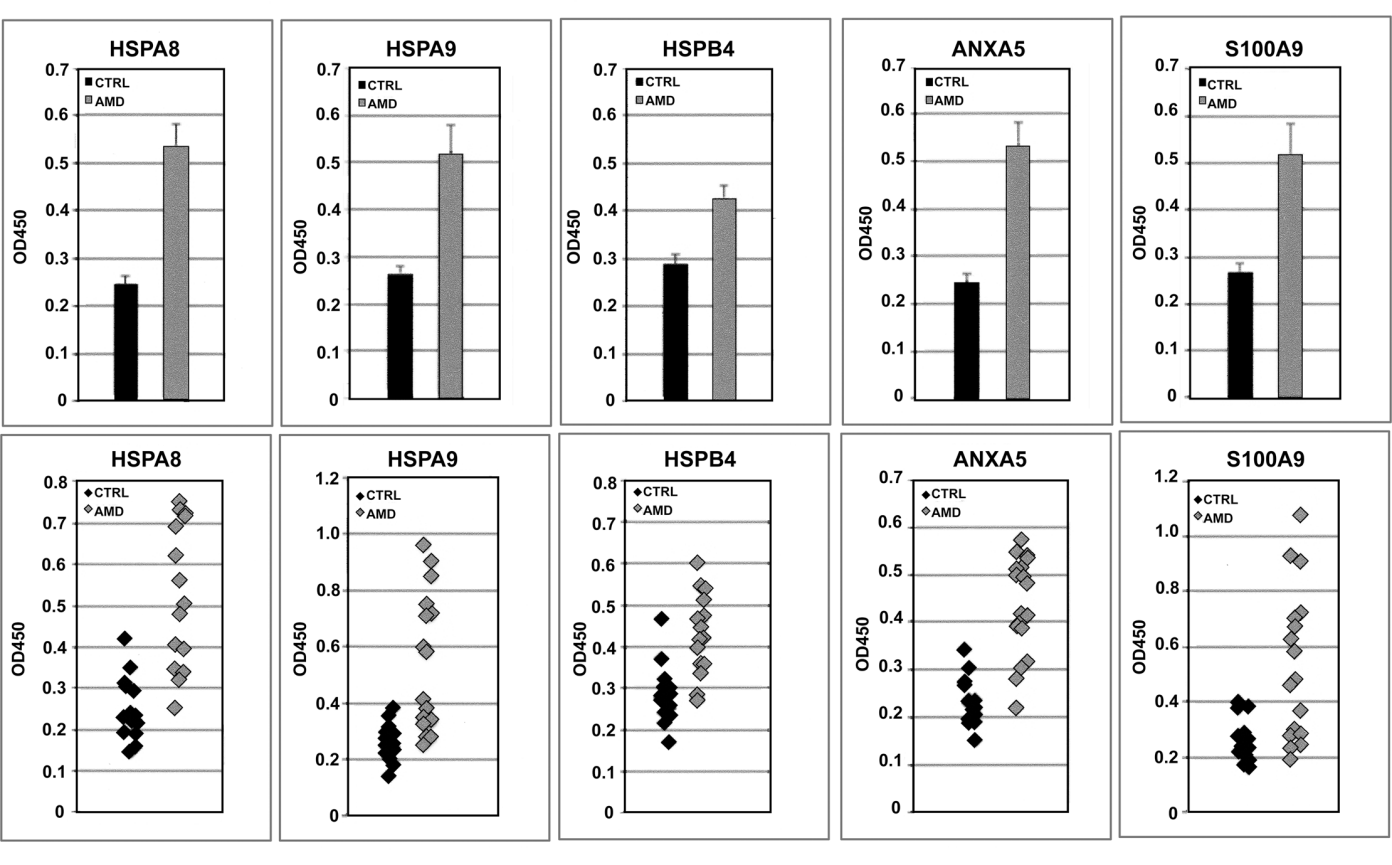
Scatterplots and bar graphs summarizing ELISA results. All autoreactivities against the five antigens identified by LC-MS/MS (HSPA8, HSPA9, HSPB4/CRYAA, ANXA5, and S100A9) were confirmed as specifically directed against these targets, and were all significantly higher in AMD than in controls.

The highly significant preferential binding of AMD sera by ELISA conclusively confirmed that HSPA8, HSPA9, HSPB4/CRYAA, ANXA5, and S100A9 are specific AAb targets in AMD (**Fig 3**): anti-HSPA8 autoreactivity was 0.54±0.04 in AMD samples (mean±SE) and 0.25±0.02 in control samples (p = 0.000003, 2-sided test for unequal variances), anti-HSPA9 autoreactivity was 0.52±0.06 in AMD samples and 0.27±0.02 in control samples (p = 0.0003, 2-sided test for unequal variances), anti-HSPB4/CRYAA autoreactivity was 0.43±0.02 in AMD samples and 0.29±0.02 in control samples (p = 0.00007, 2-sided test for equal variances), anti-ANXA5 autoreactivity was 0.44±0.03 in AMD samples and 0.24±0.01 in control samples (p = 0.0000001 2-sided test for unequal variances), and anti-S100A9 autoreactivity was 0.52±0.06 in AMD samples and 0.26±0.02 in control samples (p = 0.001, 2-sided test for unequal variances).

## Discussion

Our finding that multiple AAbs in AMD sera recognize human macular tissue antigens dovetails nicely with evidence that has accumulated over the past two decades [71–75] for a role of autoimmunity in AMD. Following seminal studies that shed light on the inflammatory and immune-mediated component of drusen formation [3, 76, 77] and proteomics studies of drusen composition [78, 79], and following the discovery linking AMD to genetic variants involved in abnormal regulation of complement-mediated inflammatory processes [28–48], the role of inflammation and of the immune system in AMD has been repeatedly confirmed [56, 58, 59, 80–87] and has become widely accepted [15–26]. It has been recently suggested that distinct AAb signatures may exist between early AMD, GA and nvAMD [59]. In summary, the concept of AMD as a disease that is at least in part immunologically driven has emerged [12–14, 24].

The partial breakdown of the blood-retinal barrier that characterizes AMD eyes also at the early stages of the diseases, the accumulation of inflammatory debris and effectors at the sub-RPE levels, and the infiltration of DCs in drusen cores all concur to creating a favorable milieu to both an autoimmune response and an active role in situ of AAbs directed against macular antigens. With regard to the specific autoreactivities that we identified, there is a large body of evidence that supports the possibility that AAbs against HSPA8, HSPA9, HSPB4/CRYAA, ANXA5 and S100A9 could be of relevance to AMD pathogenesis. The many roles and functions of these five antigens in and outside the eye for these proteins that are directly relevant to our findings and to our working hypothesis are summarized in **Table 2**, based on both information reported in the UniProt database (http://www.uniprot.org/) and additional specific references from the literature [78, 88–138]. HSPA8, HSPA9, HSPB4/CRYAA, ANXA5 and S100A9 are of particular interest due to their expression in human macular tissues, their upregulation in AMD donor eyes, their known role in autophagy, protection from oxidative stress and apoptosis, and/or immunomodulation.

**Table 2:** Summary of known roles and functions of the identified autoantigens^a^

**HSPA8** (Heat shock cognate 71 kDa protein, a.k.a. HSP70-8)
**•** Key effector of chaperone-mediated autophagy in RPE and neurons (88,89).
**•** Found in atherosclerotic plaques (90), mediates bacterial lipopolysaccharide-induced inflammatory response (91), including TNF secretion by monocytes (92), is part of the endoplasmic reticulum-associated degradation (ERAD) quality control pathway (93), and is a very important autophagy regulator (134,135).
**•** Anti-HSPA8 AAbs implicated in pathogenesis of both cancer-associated retinopathy (CAR) (94) and autoimmune hepatitis (95).
**•** Downregulator of the inflammatory response mediated by dendritic cells (DCs) and other innate immune system cells (130)
**•** In neurons, known to be involved in lysosomal degradation of α-synuclein, which accumulates in Parkinson's disease and other neurodegenerative diseases (96), and in the degradation of the amyotrophic lateral sclerosis-linked mutant SOD1 protein (97–98).
**•** SOD1 is implicated in a murine model of AMD (99,100), in which we very recently demonstrated that anti-HSPA8 AAbs develop [New et al. *Invest*. *Ophthalmol*. *Vis*. *Sci*. 2015; 56: E-Abstract 3986].
**•** Expressed in retina and RPE (101–103), in which it declines with non-pathologic aging (105), but markedly upregulated in nvAMD tissues (106).
**•** Elevated levels documented in RPE of human AMD donor eyes (107).
**HSPA9** (Stress-70 protein, mitochondrial, a.ka. GRP75)
**•** Expressed in retina and RPE (101–103), in which it declines with non-pathologic aging (105), but markedly upregulated in nvAMD tissues (106).
**•** Elevated levels documented in RPE of human AMD donor eyes (107).
**HSPB4/CRYAA** (AlphaA-crystallin)
• Anti-apoptotic, anti-oxidant protein
• Expressed in neuroretina, RPE, Bruch’s membrane and choroid (and lens) (101–103).
• Toll-like receptor 4 (TLR4)-mediated elevation of retinal HSPB4/CRYAA expression protects photoreceptors from degeneration in early stages of experimental autoimmune uveitis (104).
• Both alphaA- and alphaB-crystallins accumulate in Bruch’s membrane and subjacent choroidal connective tissue from human AMD tissues, and are abundant in drusen (78, 101, 108)
• Compromised HSPB4/CRYAA function is predicted to exacerbate oxidative stress and apoptosis in the RPE, and boosting its function is regarded as an approach for AMD and other retinal degenerative diseases (102,103, 109–111).
• Potent inhibitor of disease-causing protein aggregation in Parkinson’s disease (132)
**ANXA5 (**Annexin A5)
**•** Potent anticoagulant, highly specific ligand for phosphatidylserine (PS) via Ca2+-dependent binding, and anti-apoptotic protein involved in modulation of immune response implicated in autoimmune diseases such as antiphospholipid syndrome, rheumatoid arthritis, lupus, type 1 diabetes, and autoimmune myocharditis (112–115).
• Expressed in retina and RPE, upregulated in nvAMD tissues and, like HSPB4/CRYAA, a documented component of drusen in AMD (106, 116).
• AAbs against a related protein, Annexin II, have been found in cynomolgus monkeys with AMD (117).
**•** Stimulator of autophagy (137).
**S100A9** (S100 calcium-binding protein A9, a.k.a. calgranulin B)
**•** Ca^++^- and Zn^+^-binding protein that plays a prominent role in the regulation of inflammatory processes and immune response.
**•** Most often complexed with S100A8 to form calprotectin, which is part of a group of damage-associated molecular pattern (DAMP) molecules that trigger inflammatory responses.
• *Intracellular functions (118–127)*:
a) facilitating leukocyte arachidonic acid trafficking and metabolism;
b) modulating tubulin-dependent cytoskeleton during migration of phagocytes;
c) activating neutrophilic NADPH-oxidase;
d) induction of autophagic and apoptotic cell death via reactive oxygen species
• *Extracellular functions (118–127)*:
a) oxidant-scavenging and apoptosis-inducing activities;
b) proinflammatory roles, such as promotion of cytokine and chemokine production, leukocyte recruitment and regulation of adhesion and migration;
c) acts as a DAMP molecule and stimulates innate immune cells via binding to pattern recognition receptors (PRRs) such as TLR4 and receptor for advanced glycation end-products (AGER);
d) participates in direct selective inflammatory stimulus-dependent S-nitrosylation of multiple targets, one of which is ANXA5;
e) has a protective role as oxidant scavenger preventing exaggerated tissue damage by scavenging oxidants;
f) can act as a potent amplifier of inflammation in autoimmunity.
• Calprotectin implicated in many inflammatory and autoimmune conditions (128).
• S100 proteins are highly expressed macular protein in human donor eyes by differential proteomics studies (129), and are elevated in the BM/Ch complex of human AMD (108).

^a^ Information summarized from Uniprot and literature

The potential importance of compromised autophagy in AMD and its role in causing age-related RPE damage and loss and sub- and intra-RPE deposit accumulation has been elegantly demonstrated in recent years in rats and mice with genetically compromised small HSP-dependent autophagy pathways [131, 132, 136, 138]. In brief, autophagy is a key regulator of intracellular nutrient and energy homeostasis, affects local immune responses, and regulates both endogenous activators as well as expression of components of the inflammasome. When autophagy is compromised, a build-up in reactive oxygen species and aggregates occurs, which in turn causes NLRP3 inflammasome activation as well. The NLRP3 inflammasome is part of the pattern recognition receptors (PRRs) family devoted to recognizing pathogen-associated and danger-associated molecular patterns (DAMPs and PAMPs, respectively), and has recently emerged as a likely role player in AMD pathogenesis [131]. In conditional knock-out mice with compromised autophagy in the RPE, Yao et al. have shown a multitude of age-related AMD-like phenotypic changes, as well intraretinal and sub-RPE inflammation, Bruch’s membrane disruption, and eventual damage and loss of overlying photoreceptors [132]. Thus, AAbs directed against targets implicated in autophagy control could exacerbate AMD at least in part also via a direct autophagy-mediated mechanism.

The interplay and crosstalk between protein homeostasis, autophagy, the proteasome, and HSPs in the pathogenesis of AMD has become increasingly appreciated over the past few years [139–141] and has been recently reviewed in detail [142]. The particular role of HSPs as gatekeepers of proteolytic pathways in the RPE and the implications of the disruption of the HSP-mediated chaperone functions in the aging RPE with regard to regulation of autophagy, accumulation of oxidative stress-induced damage, protein aggregation, lipofuscinogenesis, and AMD etiology have also been reviewed [88]. In brief, HSPA8 and HSPA9 are members of the HSP70 family that is a key effector of chaperone-mediated autophagy, in the RPE as well as in neurons [88, 89]. In addition, HSP70s act as well as direct downregulators of the inflammatory response mediated by DCs and other innate immune system cells [130]. Anti-HSPA8 AAbs are implicated in the pathogenesis of both cancer-associated retinopathy (CAR) [94] and autoimmune hepatitis [95]. In neurons, HSPA8 is known to be involved in the lysosomal degradation of α-synuclein, which accumulates in Parkinson's disease and other neurodegenerative diseases [96], and in the degradation of the amyotrophic lateral sclerosis-linked mutant SOD1 protein [97, 98]. Genetic depletion of SOD1 is implicated in a murine model of AMD [99, 100], in which our group has very recently shown that anti-HSPA8 AAbs develop *before* overt AMD-like changes develop but at a stage when marked intraretinal inflammation is already present [New et al. *Invest*. *Ophthalmol*. *Vis*. *Sci*. 2015; 56: E-Abstract 3986]. Of direct relevance to AMD, HSPA8 is expressed in the retina and the RPE, in which it declines with non-pathologic aging [105], but is markedly upregulated in nvAMD tissues [106]. The specific role of HSPA8 as a chaperone-mediated autophagy regulator [88, 89] has been characterized recently, showing that it is mediated by its ATPase domain and that it is associated with induction of Akt and mTOR phosporylation [133, 134]. Elevated HSP levels have also been documented in the RPE of human AMD donor eyes [107]. Thus, AAbs against these HSPs could lead to compromised regulation of autophagy.

HSPB4, also known as CRYAA, has anti-apoptotic functions. In addition to the lens, HSPB4/CRYAA expression in the eye has been demonstrated also in nRet, RPE, BM and Ch tissues [101–103]. An elevation of retinal HSPB4/CRYAA expression that protects photoreceptors from degeneration has been reported in the early stages of experimental autoimmune uveitis [104]. Both alphaA- (CRYAA) and alphaB- (CRYAB) crystallins, two members of the small HSP family, accumulate in the BM and subjacent choroidal connective tissue from human AMD tissues, and are abundant in drusen [78, 101, 108]. Compromised HSPB4/CRYAA function is predicted to exacerbate oxidative stress and apoptosis in the RPE, and boosting its function may offer a potential treatment for AMD and other retinal degenerative diseases [102, 103, 109–111]. In Parkinson’s disease (PD), HSPB4 has been shown to be the most potent inhibitor, in an autophagy-independent fashion, of disease-causing protein aggregates induced by the C289G parkin E3-ubiquitin protein ligase (*PARK2*) mutation [135]. Thus, it can be envisioned how, in AMD, AAbs directed against HSPB4/CRYAA could compromise its functions and contribute to AMD-promoting RPE damage and loss, possibly by promoting formation of the intra- and/or extra-cellular aggregates that are seen in AMD and that can lead, independently of autophagy compromise, to NLRP3 inflammasome activation [136].

Annexin A5 (ANXA5) is an anti-apoptotic protein involved in the modulation of immune response repeatedly implicated in autoimmune diseases such as antiphospholipid syndrome, rheumatoid arthritis, lupus, type-1 diabetes, and autoimmune myocharditis [112–115]. Of specific relevance to AMD, ANXA5 is expressed in retina and RPE, is upregulated in nvAMD tissues and, like HSPB4, is a documented component of drusen in AMD [106, 116]. Furthermore, AAbs against a related protein, Annexin II, have been found in cynomolgus monkeys with AMD [117]. Interestingly, ANXA5 has been shown to be an autophagy stimulator [137]. Thus, much like the anti-HSPA8, A9 and B4 AAbs, also anti-ANXA5 AAbs could in theory contribute to AMD pathogenesis at least in part via an autophagy-mediated mechanism.

Lastly, S100 calcium-binding protein A9 (S100A9), also known as calgranulin B, plays a prominent role in the regulation of inflammatory processes and immune response. S100A9 is most often complexed with S100A8 to form calprotectin, which too is part of a group of DAMP molecules that trigger inflammatory responses and stimulate innate immune cells via PRR binding that is implicated in a plethora on inflammatory and autoimmune conditions [128]. Individually, S100A9 has many intracellular and extracellular functions, including direct selective inflammatory stimulus-dependent S-nitrosylation of multiple targets, one of which is ANXA5, and acting as a potent amplifier of inflammation in autoimmunity [118–127]. Of direct relevance to AMD, S100 proteins have been recently identified as a highly expressed macular protein in human donor eyes by differential proteomics studies [129], and are elevated in the BM/Ch complex of human AMD [108]. Of further interest, calprotectin has been shown to induce both apoptosis and autophagy via a ROS-mediated cross-talk between mitochondria and lysosomes, a dual property that is shared also by other factors [122]. Thus, several mechanisms via which anti-S100A9 AAbs could contribute to AMD pathogenesis could be at play.

The hypothesized connection between the autoreactivities that we detected and mechanisms linked to autophagy, NLRP3 inflammasome activation, protection from oxidative stress and apoptosis, and/or immunomodulation is well supported by a large body of published evidence. However, we do not know yet whether the AAbs detected in our study have either partially causal and/or contributory roles in AMD pathogenesis via these potential mechanisms, and/or which of them may be preeminent. With this limitation in mind, the pathogenicity of anti-retinal AAbs has already been established in patients with cancer-associated retinopathy (CAR), a form of secondary autoimmune retinopathy (AIR), as well as other forms of primary AIR and neuro-retinopathy (AINR) [143–153]. In these conditions, many distinct AAbs are found, and can coexist in the same patient. The similarity in IR patterns between AMD and AINR/CAR, noted already by Adamus et al. [59], raises the intriguing possibility that AMD may share at least in part some fundamentally similar pathogenic mechanism with these autoimmune retinal conditions [143–153].

Another caveat to our study is that the subset of participants we chose to confirm the IDs obtained via LC-MS/MS, and quite possibly even all of the 131 participants with AMD in our study sample, were in all likelihood not fully representative of all the possible reactivities that could be meaningful to AMD. Some other reactivities may have been missed by testing only a subset of samples, and others that did not meet the B-H multiple comparison correction statistical method used in our analyses may have actually achieved significance with an even larger data set. Thus, additional and potentially important reactivities that were not identified in our study likely exist in AMD (see, e.g., Adamus et al. [59]). As part of our ongoing effort to elucidate further the role of autoimmunity in AMD, we will continue to expand and refine our search for additional demonstrable reactivities in larger and/or different participant serum sample collections in the future.

Our study benefited from the fact that all of our subjects were carefully examined and graded with respect to their fundus findings, and were chosen to be free of ocular or systemic conditions of inflammatory and autoimmune nature and/or that could potentially confound our study. Furthermore, the use of human tissue antigens, and specifically macular ones, to test sera for reactivity supports the nature of the detected autoantigens as bona fide macular ones. In addition, the confirmation of our results by ELISA demonstrates the presence of AAbs directed against HSPA8, HSPA9, HSPB4/CRYAA, ANXA5, and S100A9 – antigens that are related to one another via their implication in protection from oxidative stress and apoptosis immunomodulation, and especially autophagy, a common theme shared by all targets identified by our investigation. This finding suggests that the detected AAbs may all contribute, from different angles, to compromised autophagy and contribute to NLRP3 inflammasome activation in AMD [131]. Our study design allowed us to gain information about the role of autoimmunity across the entire AMD disease spectrum. However, by focusing particularly on early AMD, we were able to gain particular insight on the role that AAbs may play early on in AMD pathogenesis. Thus, without suggesting that AMD could be a primary autoimmune condition like CAR and AINR, we can confidently conclude that our findings support the notion that the observed AAbs are unlikely to be mere after-the-fact biomarkers of advanced degeneration that has already occurred. Rather, we suggest that they represent valuable biomarkers of highly plausible pathogenic potential. As it has been already done for CEP [56, 58, 80, 82, 84, 85] and for similar AAb findings in glaucoma [154–156], additional studies aimed at verifying the direct pathogenicity of these AAbs and the mechanisms via which the targeted proteins could participate in AMD biogenesis are now necessary, and represent a key ongoing research focus of our laboratories.

## Supporting Information

S1 Fig Schematic step-by-step representation of the methodology used to harvest full-thickness macular tissue punches and prepare the whole-macular lysates. See S1 Supplemental Methods text for detailed explanations of the individual steps.
(PDF)

S2 Fig Example of gel bands captured for analysis in ImageJ and corresponding densitometric band plots generated by ImageJ64. Bands were measured from “peak darkness” (shown as the trough in the densitometric plots) to baseline. Measured values were expressed as the relative fractional darkness of a reference band (not shown) on the same gel lane, whereby the apparent differences that could at times exist in the background were identical within each gel lane, making it unnecessary to account for different background intensities between different gel lanes. Thus, e.g., a band that appeared as dark as the reference band would usual give a 0.98 darkness ratio. One that seemed about half as dark, would correspond roughly to a 0.50 ratio, and so on. Note how every element in the cropped gel box is analyzed by ImageJ, including a double peak for the hand-written “37” shown above or a sharp, square-looking spike for the white background captured on the right-hand side of each gel. Note also that ImageJ offers the possibility to plot the densitometric data inverted as peaks instead of troughs at the discretion of the user.
(PDF)

S3 Fig Representative MS/MS spectra for the peptides matched to proteins identified in this study (see Table 1). The spectra were produced via collision-induced dissociation (CID) of the corresponding mass-selected precursor ions in the ion trap mass spectrometer. The MS/MS spectra contain sequence-determining product ions of the b- (shown in red) and y-series (shown in blue).
(PDF)

S1 Supporting Information Supplemental Methods. (DOCX)

S1 Table Immunoreactivity classification criteria used to grade the Western blot bands exposed to chemiluminescent substrate and imaged at 5, 15, 30 seconds (sec). Scores (0–5) represent IR intensity of increasing intensity.
(PDF)
