# Supplementary material for: Circulating Autoantibodies in Age-Related Macular Degeneration Recognize Human Macular Tissue Antigens Implicated in Autophagy, Immunomodulation, and Protection from Oxidative Stress and Apoptosis
Source: PLoS One. 2015 Dec 30;10(12):e0145323. doi: 10.1371/journal.pone.0145323 (PMC4696815; doi:10.1371/journal.pone.0145323)
Supplement: S1 Fig — See S1 Supplemental Methods text for detailed explanations of the individual steps. (PDF) [file pone.0145323.s001.pdf]

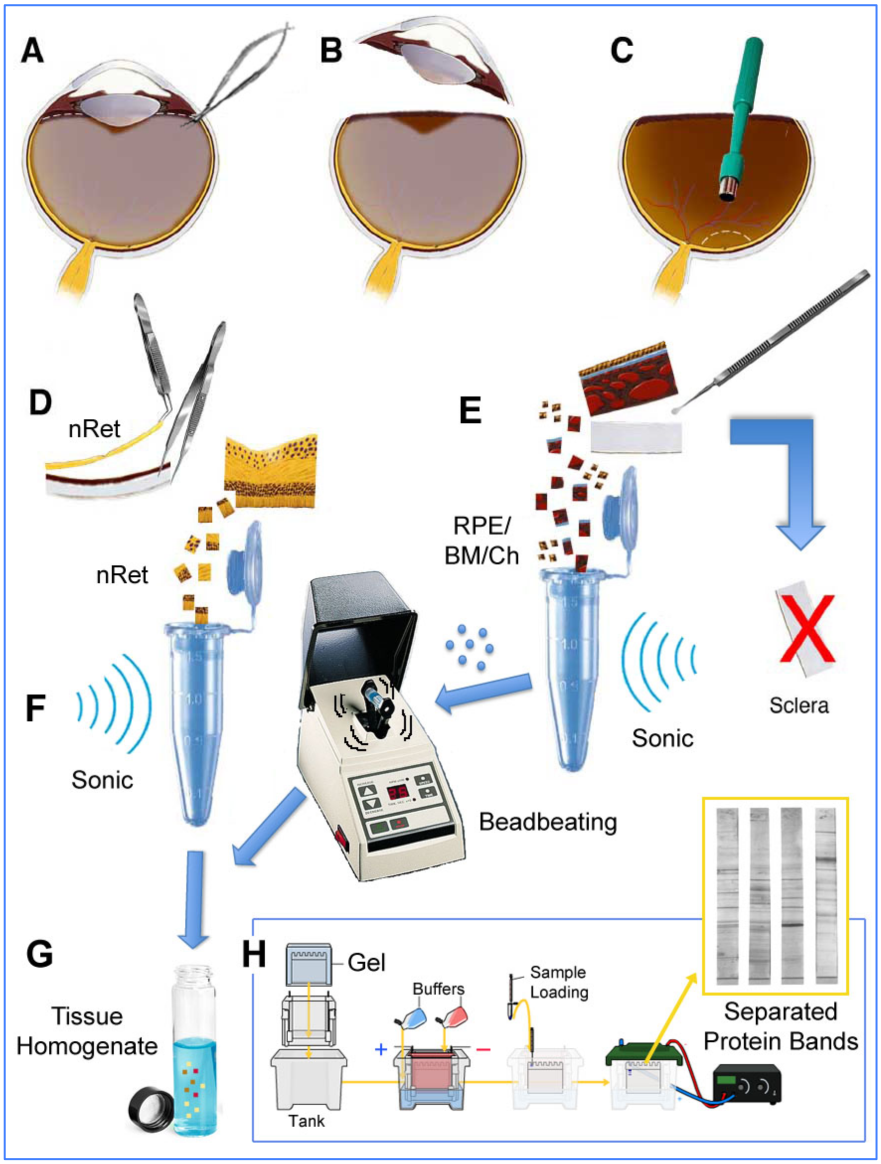

S1 Fig. Schematic step-by-step representation of the methodology used to harvest full-thickness macular tissue punches and prepare the whole-macular lysates. See S1 Supplemental Methods text for detailed explanations of the individual steps.
