# Supplementary material for: Circulating Autoantibodies in Age-Related Macular Degeneration Recognize Human Macular Tissue Antigens Implicated in Autophagy, Immunomodulation, and Protection from Oxidative Stress and Apoptosis
Source: PLoS One. 2015 Dec 30;10(12):e0145323. doi: 10.1371/journal.pone.0145323 (PMC4696815; doi:10.1371/journal.pone.0145323)
Supplement: S2 Fig — Bands were measured from “peak darkness” (shown as the trough in the densitometric plots) to baseline. Measured values were expressed as the relative fractional darkness of a reference band (not shown) on the same gel lane, whereby the apparent differences that could at times exist in the background were identical within each gel lane, making it unnecessary to account for different background intensities between different gel lanes. Thus, e.g., a band that appeared as dark as the reference band would usual give a 0.98 darkness ratio. One that seemed about half as dark, would correspond roughly to a 0.50 ratio, and so on. Note how every element in the cropped gel box is analyzed by ImageJ, including a double peak for the hand-written “37” shown above or a sharp, square-looking spike for the white background captured on the right-hand side of each gel. Note also that ImageJ offers the possibility to plot the densitometric data inverted as peaks instead of troughs at the discretion of the user. (PDF) [file pone.0145323.s002.pdf]

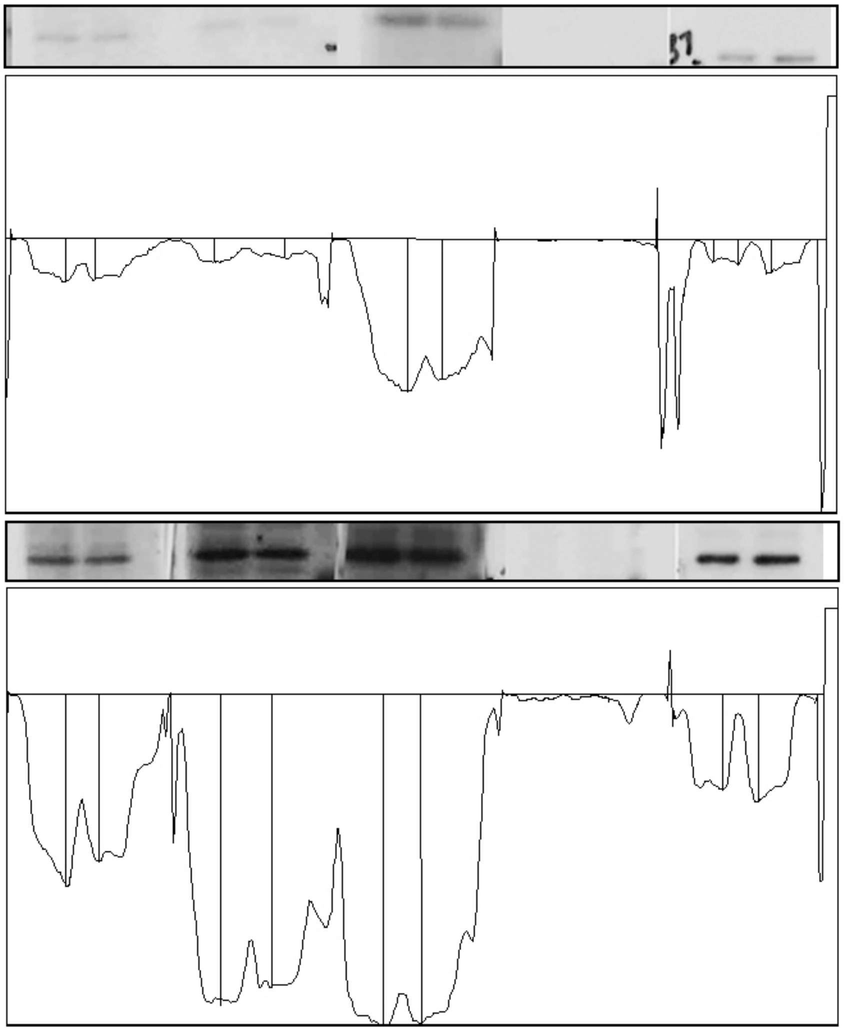

S2 Fig. Example of gel bands captured for analysis in ImageJ and corresponding densitometric band plots generated by ImageJ64. Bands were measured from “peak darkness” (shown as the trough in the densitometric plots) to baseline. Measured values were expressed as the relative fractional darkness of a reference band (not shown) on the same gel lane, whereby the apparent differences that could at times exist in the background were identical within each gel lane, making it unnecessary to account for different background intensities between different gel lanes. Thus, e.g., a band that appeared as dark as the reference band would usual give a 0.98 darkness ratio. One that seemed about half as dark, would correspond roughly to a 0.50 ratio, and so on. Note how every element in the cropped gel box is analyzed by ImageJ, including a double peak for the hand-written “37” shown above or a sharp, square-looking spike for the white background captured on the right-hand side of each gel. Note also that ImageJ offers the possibility to plot the densitometric data inverted as peaks instead of troughs at the discretion of the user.
