# Supplementary material for: Circulating Autoantibodies in Age-Related Macular Degeneration Recognize Human Macular Tissue Antigens Implicated in Autophagy, Immunomodulation, and Protection from Oxidative Stress and Apoptosis
Source: PLoS One. 2015 Dec 30;10(12):e0145323. doi: 10.1371/journal.pone.0145323 (PMC4696815; doi:10.1371/journal.pone.0145323)
Supplement: S3 Fig — The spectra were produced via collision-induced dissociation (CID) of the corresponding mass-selected precursor ions in the ion trap mass spectrometer. The MS/MS spectra contain sequence-determining product ions of the b- (shown in red) and y-series (shown in blue). (PDF) [file pone.0145323.s003.pdf]

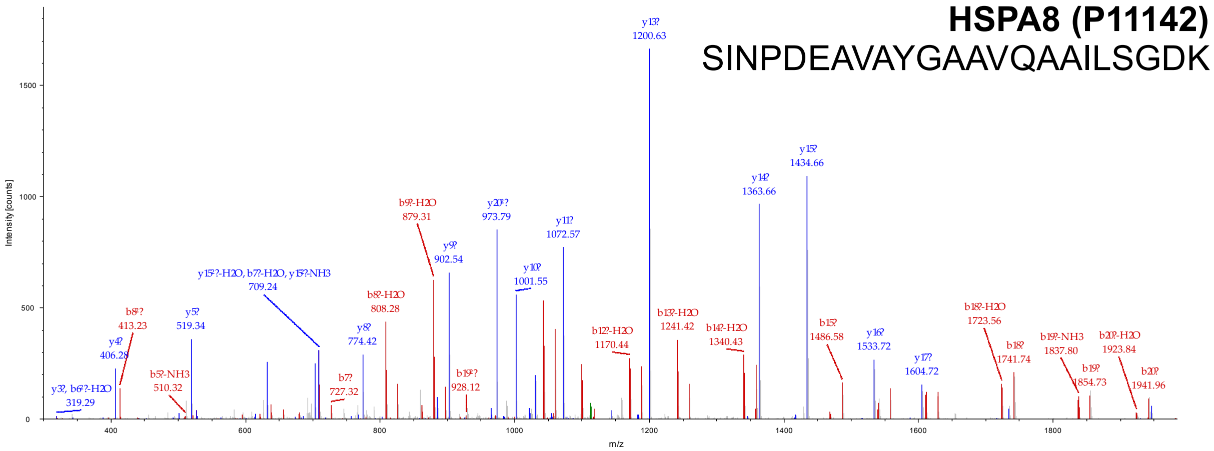
HSPA8 (P11142)
SINPDEAVAYGAAVQAAILSGDK
Intensity [counts]
m/z

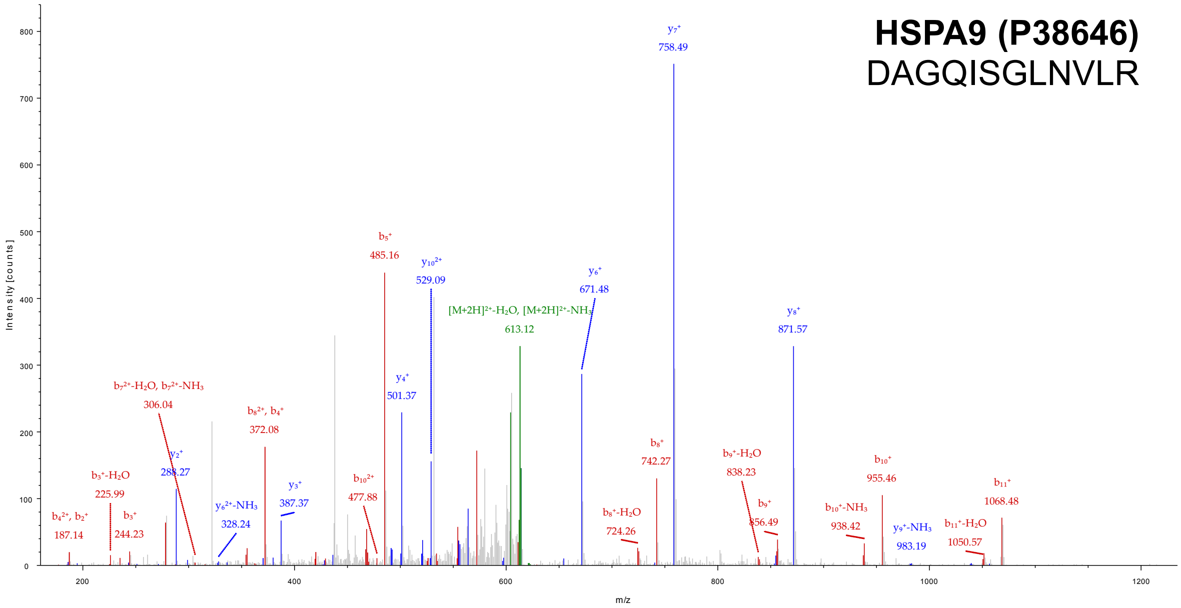
HSPA9 (P38646)
DAGQISGLNVLR
Intensity [counts]
m/z

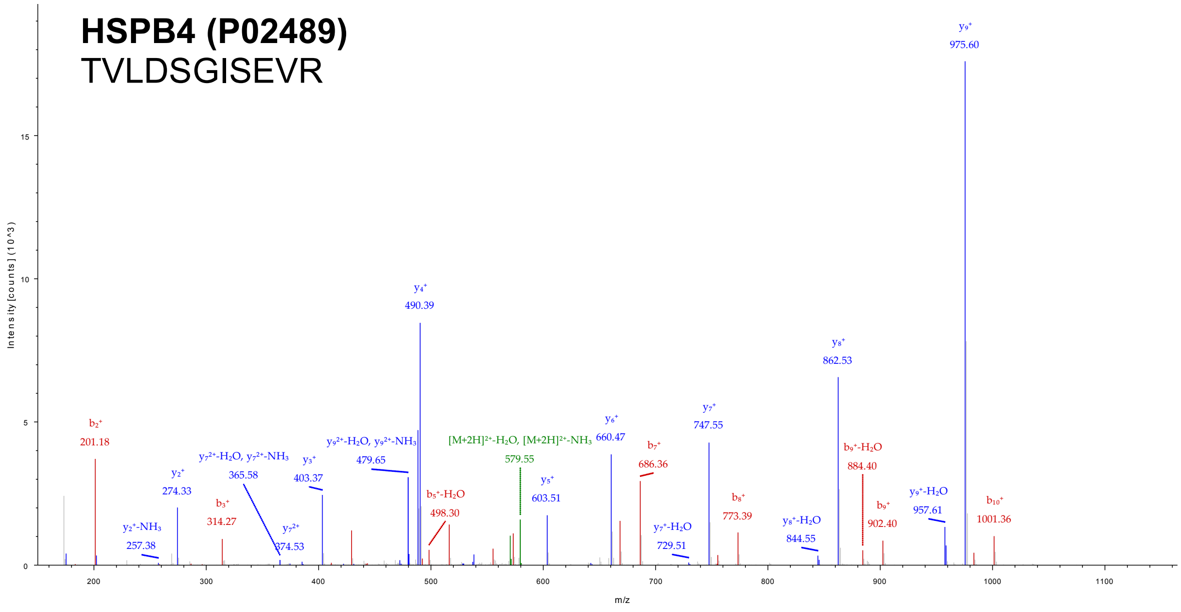
HSPB4 (P02489)
TVLDSGISEVR
Intensity [counts] (10^3)
m/z

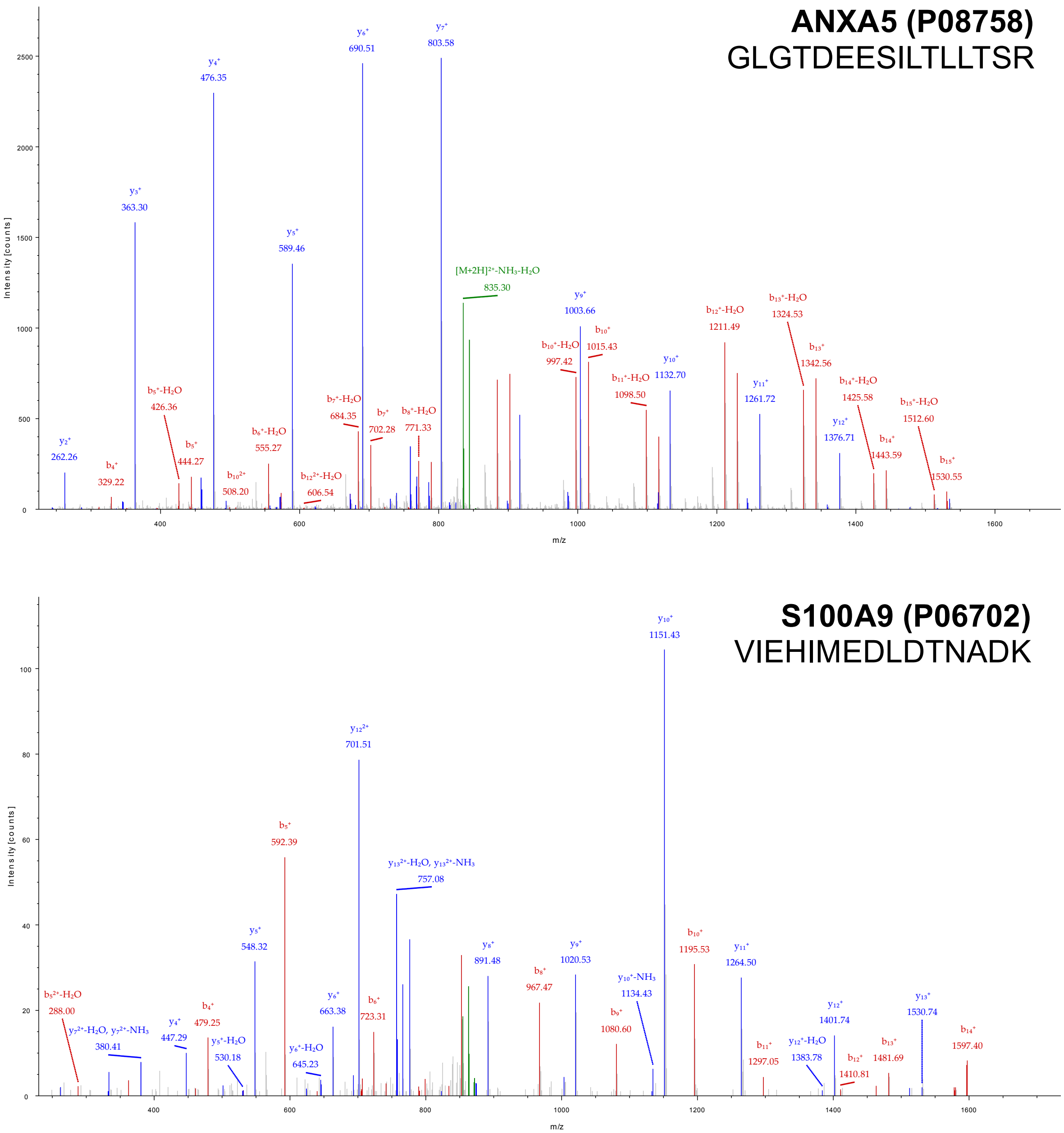

S3 Fig. Representative MS/MS spectra for the peptides matched to proteins identified in this study (see Table 1). The spectra were produced via collision-induced dissociation (CID) of the corresponding mass-selected precursor ions in the ion trap mass spectrometer. The MS/MS spectra contain sequence-determining product ions of the b- (shown in red) and y-series (shown in blue).
