# Supplementary material for: Circulating Autoantibodies in Age-Related Macular Degeneration Recognize Human Macular Tissue Antigens Implicated in Autophagy, Immunomodulation, and Protection from Oxidative Stress and Apoptosis
Source: PLoS One. 2015 Dec 30;10(12):e0145323. doi: 10.1371/journal.pone.0145323 (PMC4696815; doi:10.1371/journal.pone.0145323)
Supplement: S1 Supporting Information — (DOCX) [file pone.0145323.s004.docx]

**S1 Supporting Information**

**Supplemental Methods**

**Details about participant population**

Our study involved primarily participants in the Health ABC Age-Related Maculopathy Ancillary (ARMA) Study, a NEI-funded ancillary study to the NIA-sponsored prospective study of aging Health ABC, taking place in Memphis, TN and Pittsburgh, PA, USA. Only Memphis subjects participated in our ARMA Study.

Since advanced AMD was underrepresented by study design in both ARMA and Health ABC, we subsequently enriched our sample with advanced AMD cases from the general population of Memphis, TN, from the practices of the Principal Investigator (AI) and other collaborators. The retinal status of all 362 ARMA study participants was assessed by the PI. Retinal conditions of original ARMA participants were independently also verified by an experienced fundus photography masked grader from the University Wisconsin Fundus Photography Reading Center. The PI adjudicated discrepancies between clinical assessment of ARMA participants and the Reading Center (<10% of cases) by re-grading fundus images in masked fashion. Retinal status in the advanced AMD subjects recruited from the general population was verified via direct examination, review of records, and evaluation of retinal color photos, optical coherence tomography and/or autofluorescence. Retinal findings for all participants were classified with the original AREDS grading system, based on the original approved ARMA Study protocol.[^1^](#_ENREF_1)

**Detailed human donor tissue harvesting methodology**

Sera were assessed for reactivity against normal human tissue homogenates comprising neuroretina (nRet), RPE, BM and choroid (Ch), harvested exclusively from the macular region of human donor eyes (≥60 yo) collected by the Mid-South Eye Bank and the National Disease Research Interchange (NDRI, Philadelphia, PA) and provided to us as anonymous specimens inclusive only of age, gender, cause of death, time to collection, ocular and systemic history. None of the donor eyes had a preexisting history of diabetes/diabetic retinopathy or of glaucoma to minimize confounding from these conditions (exclusion criteria). As illustrated in **S1 Fig.**, after resecting (**S1A**) and removing (**S1B**) the anterior segment of the human donor eyes, the vitreous gel was gently removed, the remaining posterior pole were further dissected and 10 mm full-thickness punches were obtained from the macular region with a dermal biopsy punch (Miltex, York, PA) (**S1C**). The isolated tissues from the macula areas were ground and sonicated (**S1D-F**). Due to the high content of collagen fibers in the BM/Ch layers, the BM/Ch/RPE macular punch was also subjected to bead beating (**S1E**). Homogenized tissues were subsequently pooled together (**S1G**) to yield a “whole macular homogenate” inclusive of nRet, RPE, BM and Ch macular antigens from the entire full-thickness punch. The submacular scleral button (see red “X” in **S1Fig**) was discarded in all cases.

**Detailed Western blot methodology**

Macular homogenates (15 ug) were pre-cleared with protein A/G agarose beads (Pierce Cat#20423), then loaded into 12% precast SDS-PAGE gel (**S1H Fig.**) and resolved using a Mini-PROTEAN Tetra Cell apparatus (Bio-Rad Laboratories) run at 150 V for approximately 1.5 hrs. The proteins were then transferred to nitrocellulose membranes. Transfer was confirmed by visualization with Ponceau-S staining.

The nitrocellulose membranes were cut into strips, blocked with 5% milk powder in Tris-Buffered Saline with 2% Tween 20 (TBS-T) for 1 hour, and incubated with human sera, diluted 1:150 in 2.5% milk in 2% TBS-T, overnight at 4ºC. Membranes were washed and incubated with a 1:30,000 dilution of goat anti-human IgG conjugated to horseradish peroxidase (Southern Biotech), in 2.5% milk in 2% TBS-T, for 1 hr at room temperature. Membranes were washed after incubations with 2% TBS-T and 1x TBS for 10 minutes. Strips were then incubated for 2 min in the dark with approximately 400μl of Plus-ECL Enhanced Chemiluminescence Substrate (Pierce, Life Technologies, Grand Island, NY), transferred to an autoradiography cassette, and exposed to film in a dark room.

**Detailed immunoreactivity analysis**

The kilo-Dalton (kDa) value for each IR band on our WB gels was determined using measurements from the standard SDS-PAGE ladder (BioRad) run on the same gel. In each region of the ladder, 1 kDa was calculated to correspond to a given distance in millimeters, thus the distance of each band from the nearest marker could be used to obtain an approximate molecular weight value.

The presence or absence of reactivity, and its relative level of intensity were recorded in 2-kDa intervals for each affected individual (n=131) and control (n=231). Once the molecular weights were determined, the relative intensity of reactivity was recorded using a 6-step (grades 0-5) classification based on visual inspection across three exposure levels (5, 15 and 30 sec). The details of this classification method are illustrated in **S1 Tab**.

The utilization of this 3-exposure level method allowed us to capture both faint reactivities that were seen only after 30-sec, and reactivities seen already after only a 5-sec exposure. Although resulting in overexposed images for some gels, especially at 30-sec which could obscure the visualization of certain bands seen with shorter exposures, this approach allowed us to reveal fainter reactivity against some antigens that were unique to AMD samples otherwise not seen at shorter exposures in many instances. These limitations to the approach that we used may have under-detected, but certainly did not over-detect reactive bands.

Based on these methods, 100 data points (IR scores every 2-kDa) were generated for each serum sample, i.e., 13,100 data points for the AMD serum sample group and 23,100 data points for the control serum sample group. This large data set was analyzed as described in the main body of the manuscript.

After the completion of the grading effort (in 2012), a subset of the WBs has been more recently regarded utilizing ImageJ64 v.1.48 for Mac analysis software, freely available on the web at <http://imagej.nih.gov/ij/>, to obtain more quantitative estimates of the reactivity levels and an indirect verification of the accuracy of the grading method. In all cases, reactivity was expressed relatively to a reference band for each WB lane, which were always ran in duplicate. To do this, WB gels were scanned at high resolution as image files, and the reactivity of individual bands was quantitated using the “Plot Lanes” function in ImageJ64. Then, the peaks-and-valleys plots resulting from the densitometric analysis of the bands were inspected, the baseline of the reactivity vs. gel background was determined and a horizontal line was drawn across the bands to be graded on the gel sets. The lowest trough of individual reactivities (oriented downwards as measure of band darkness) was measured. When a band exhibited a split trough, the average of each trough was used to quantitate reactivity. An example of one such set of bands and their respective densitometric measurements with ImageJ64 is shown is **S2 Fig.**

In the examples illustrated in **S2 Fig.**, the same bands from an identical gel set imaged after 5-sec (top) and 15-sec (bottom) exposures are presented to show how the ImageJ readouts were used to compare them to the 6-step, visual inspection-based method. Comparisons of various other representative bands from our large gel data set showed that grade 1 bands were read in ImageJ as (correctly) absent after 5- or 15-sec exposures, and had relative densitometric values ranging anywhere between 0.10 and 0.55 after 30-sec exposures. Grade 2 bands were read in ImageJ as (correctly) absent after 5-sec exposures, showed densitometric values ranging between 0.09 and 0.16 after 15-sec, and were read as typically ranging between 0.37 and 0.53. Grade 3 bands yielded usually densitometric values ≤0.18 after 5-sec exposures, between 0.19 and 0.57 after 15-sec exposures, and ≥0.83 after 30-sec exposures. Examples of grade 4 bands re-analyzed with ImageJ exhibited densitometric values around 0.13-0.26 after 5-sec exposures, 0.49-0.95 after 15-sec exposures, and 0.82-0.98 after 30-sec exposures. Lastly, grade 5 bands were read in ImageJ as consistently ≥0.50 after 5-sec exposures and consistently around 0.98-0.99 already after 15-sec (and 30-sec) exposures.

Thus, after comparison with the ImageJ-based method, it was apparent that the 6-step grading system was robust and remained entirely adequate, although far more labor-intensive, to account for different levels of reactivities at the different exposure times and was best suited for the statistical analyses to be conducted. Thus, all analyses presented in this manuscript are based on the original 6-step grading system that we had developed. However, should we were to repeat a similar study or add more samples in the future, the grading could be performed faster and in a more quantitative way with the semi-automated ImageJ-based method – or, perhaps better yet, it could be performed running gels differently and quantify them in fully automated fashion with an Odyssey CLx infrared imaging system (Li-Cor, Lincoln, NE) system or equivalent technology. The latter was not possible for our serum sample set because the technology became available to us too late to allow for re-running and re-grading all serum samples, which are also limited in quantity, especially for the Health ABC participants. For this reason, not only all the re-gradings, but also IPs and ELISA was performed exclusively on participants collected from the general population, for whom serum aliquots available to us were more abundant.

**Detailed 2D Gel Electrophoresis (2D-GE) Methods**

Human macular homogenates (150 μg) solubilized in rehydration buffer were immunoprecipitated overnight with human sera at 1:100. The resulting immune complex was then adsorbed to IgG-conjugated Protein A/G agarose beads, boiled and loaded onto a precast IPG strip with a linear pH 3-10 gradient (Amersham) and the strip was rehydrated for 12 hr. First-dimensional electrophoresis was performed on an IPGphor isoelectric focusing system (Amersham) under the following conditions: 100 V for 2 h, 500 V for 1 h, 1000 V for 1 h, and 8000 V up to 90,000 Vh. After isoelectric focusing separated proteins by their isoelectric point (pI) across the pH gradient of the strip, the strips were stored at -80ºC. Before SDS-PAGE 2DGE, the strips were equilibrated for 10 min in reducing buffer containing 6M urea, 1.5 M Tris-HCl (pH 8.8), 30% v/v glycerol, 2% w/v SDS, a trace of bromophenol blue, and 2.0% w/v DTT, and then for 10 min in alkylating buffer containing 2.5% w/v iodoacetamide. The reduced and alkylated strips were transferred onto vertical 12% SDS-PAGE gels and fixed in place with 1% agarose. 2DGE was performed on a Protean Plus Dodeca Cell electrophoresis apparatus from Bio-Rad (Hercules, CA, USA) for 430 min at constant voltage of 200 V. The final gel was stained in SYPRO Ruby (Molecular Probes) with gentle agitation for 3 hrs to overnight in the dark and washed in 10% methanol and 7% acetic acid. Proteins were detected using a laser scanner with excitation 488 and 555 nm bandpass emission filter and gel spots were quantified with Progenesis protein quantification software (Nonlinear Dynamics). Differences in spot intensity between control and AMD sera were quantified automatically by the software by one-way analysis of variance (ANOVA).

**Detailed Liquid Chromatography Tandem Mass Spectrometry (LC-MS/MS) Methods**

The proteins in the 2D-GE spots of interest were digested with trypsin as reported previously,[^2^](#_ENREF_2) [^3^](#_ENREF_3) with reduction and alkylation steps included in the protein processing workflow. The digests were purified with a ZipTip C18 (Millipore) using manufacturer’s procedures. The LC-MS/MS analyses were performed with an LTQ linear ion trap mass spectrometer (Thermo Scientific) interfaced with a Famos/Ultimate nanoflow LC system (Dionex). The nano LC separations were performed with a fused silica capillary column/spray needle (15 cm length, 75 µm i.d., New Objective) packed in-house with reverse-phase particles (MAGIC C18 from Michrom Bioresources) using a linear gradient from 0% to 90% mobile phase B at a flow rate of 200 nL/min. Mobile phase B was 10% water/90% methanol/0.05% formic acid; mobile phase A was 98% water/2% methanol/0.05% formic acid. For acquisition of LC-MS/MS data, the mass spectrometer was operated in the data-dependent mode; each cycle consisted of an MS followed by MS/MS of seven most abundant precursor ions. The LC-MS/MS datasets were used to interrogate the UniProt protein sequence database (subset of human proteins) using the SEQUEST HT search engine (Proteome Discoverer 1.4 software suite, Thermo Scientific). The search parameters were: full trypsin specificity; dynamic modification of oxidized methionine; and static modification of carbamidomethylated cysteine. The search results were filtered with the Percolator [^4^](#_ENREF_4) algorithm, which assigns statistical probability values – q-value and PEP value – to each peptide spectrum match (PSM). Percolator uses a decoy database derived from the database used for the search. In this study, we used a threshold value of q = 0.01, which corresponds to an FDR of 1 % for peptide match assignment. Only proteins with two or more peptide matches were accepted. All spectra for the matched peptides were manually validated.

**Supplemental References**

**1.** Age-Related Eye Disease Study Research Group. The Age-Related Eye Disease Study system for classifying age-related macular degeneration from stereoscopic color fundus photographs: the Age-Related Eye Disease Study Report Number 6. *Am J Ophthalmol.* Nov 2001;132(5):668-681.

**2.** Pabst MJ, Pabst KM, Handsman DB, Beranova-Giorgianni S, Giorgianni F. Proteome of monocyte priming by lipopolysaccharide, including changes in interleukin-1beta and leukocyte elastase inhibitor. *Proteome Sci.* 2008;6:13.

**3.** Giorgianni F, Cappiello A, Beranova-Giorgianni S, Palma P, Trufelli H, Desiderio DM. LC-MS/MS analysis of peptides with methanol as organic modifier: improved limits of detection. *Anal Chem.* 12/1/2004 2004;76(23):7028-7038.

**4.** Käll L, Storey JD, MacCoss MJ, Noble WS. Assigning significance to peptides identified by tandem mass spectrometry using decoy databases. *J Proteome Res.* 2007;7(01):29-34.
