# Supplementary material for: Circulating Autoantibodies in Age-Related Macular Degeneration Recognize Human Macular Tissue Antigens Implicated in Autophagy, Immunomodulation, and Protection from Oxidative Stress and Apoptosis
Source: PLoS One. 2015 Dec 30;10(12):e0145323. doi: 10.1371/journal.pone.0145323 (PMC4696815; doi:10.1371/journal.pone.0145323)
Supplement: S1 Table — Scores (0–5) represent IR intensity of increasing intensity. (PDF) [file pone.0145323.s005.pdf]

S1 Table. Immunoreactivity classification criteria used to grade the Western blot bands exposed to chemiluminescent substrate and imaged at 5, 15, 30 seconds (sec). Scores (0-5) represent IR intensity of increasing intensity.

SCORE (0-5)	IMMUNOREACTIVITY (IR)	EXPOSURE TIME		
		**5 sec**	**15 sec**	**30 sec**
**0**	**No reactivity**	No bands	No bands	No bands
**1**	**Trace positive**	No bands	No bands	Faint to moderate band intensity
**2**	**Weakly positive**	No bands	Faint band intensity	Up to moderate band intensity
**3**	**Moderately positive**	Faint band intensity	Moderate band intensity	Up to strong band intensity [a]
**4**	**Strongly positive**	Moderate band intensity	Up to strong band intensity [a]	Greater intensity than 15 sec [b]
**5**	**Maximal reactivity**	Strong band intensity [a]	Greater intensity than 5 sec [b]	Greater intensity than 15 sec [b]

[a] Approximately identical to a reference band in each gel lane

[b] Typically saturating intensity seen at this exposure
